# Impact of Scoring First on Match Outcome in the Chinese Football Super League

**DOI:** 10.3389/fpsyg.2021.662708

**Published:** 2021-05-28

**Authors:** Tianbiao Liu, Antonio García-de-Alcaraz, Hai Wang, Ping Hu, Qiu Chen

**Affiliations:** ^1^College of P.E. and Sports, Beijing Normal University, Beijing, China; ^2^Lab of Performance Analysis and Motor Learning, Beijing Normal University, Beijing, China; ^3^Department of Education, University of Almería, Almería, Spain; ^4^LFE Research Group, Universidad Politécnica de Madrid, Madrid, Spain; ^5^Microsoft, Beijing, China; ^6^National School of Development, Peking University, Beijing, China

**Keywords:** team sports, performance analysis, score, match status, situational variables, goal

## Abstract

The aim of this study was to explore the effect of scoring first on match outcomes in the Chinese Football Super League (CSL). A total of 1,116 matches in which at least one goal was scored from the 2014 to 2018 seasons were collected. Match outcomes, absolute goal differences, the minute of the first goal, match locations, and teams’ budgets were analyzed. A team’s budget was measured in terms of a team’s value at the beginning of the season, and teams were clustered into two groups (high and low budget with means of 50.77 and 13.77 million dollars, respectively). A descriptive analysis was conducted, and two generalized linear models (a multinomial logit model and a Poisson model; *p* < 0.05) were applied. The results showed a favorable outcome for the team that scored first both in match outcome and goal difference. Regarding the teams that scored first, 66.31% won their matches, 20.70% achieved a draw, and 12.99% lost. Specifically, home teams were more likely to win (13.42%) and less likely to lose (9.52%) or draw (3.90%) than away teams. Home teams also had a higher likelihood of obtaining a larger goal difference. Higher budget teams were more likely to win (14.90%) and less likely to lose (9.75%) or draw (5.14%) than low-budget teams. Additionally, for each minute, the team scores closer to the end of the match, and the average probability of winning increased by 0.0028. These findings can guide the strategies of coaches in different match scenarios according to the match location and the opponent’s quality.

## Introduction

Football is a time-dependent sport whose result depends on comparing the goals of the two participating teams at the end of the match (Hughes and Franks, 2004). However, most matches ended with no more than three goals (Anderson and Sally, 2013). Therefore, every goal is critical and influences the course of the game (Ferreira, 2013). In many team sports, scoring first has already been proven to be crucial to the match outcome (Jones, 2009). Moreover, when the match approaches the end, the result is nearly determined (Altarriba-Bartés et al., 2020). Overall, scoring first has a strong influence on match outcome (Bloomfield et al., 2005). Across worldwide football tournaments, scoring first has been shown to be an effective predictor of the final outcome in the 2018 FIFA (Fédération Internationale de Football Association) World Cup (71.40%; Vergonis et al., 2019), the six major football tournaments in the world (71.17%; Leite, 2015), the Greek league in the 2006–2007 (71.43%; Armatas et al., 2009b) and 2007–2008 seasons (74.20%; Armatas et al., 2009a), and the La Liga (Spanish League) from the 2005 to 2010 seasons (79.32%; Molinuevo and Bermejo, 2012).

The team that scores first breaks the competitive balance and forms a new match status. This shift of match status may lead to changes in match strategies, tactics, and styles of play for each team (Taylor et al., 2008; Lago, 2009; Almeida et al., 2014) and may even alter players’ psychological states and, hence, influence their performance (Bar-Eli et al., 2006; Leite and Barreira, 2014). For instance, several studies have found a greater ball possession time among losing teams (Lago-Peñas and Dellal, 2010; Taylor et al., 2010; Ruiz-Ruiz et al., 2013). Although scoring first appears to be a key event in winning a match, sometimes the team who scores first loses. The complex interaction between multiple factors is a plausible reason that should be investigated (Garganta, 1997). In this sense, the situational variables have been considered. Thus, match location has been considered to be a strong indicator (Molinuevo and Bermejo, 2012; Tenga, 2013; Pratas et al., 2016). Home teams have better technical and tactical performance (Sasaki et al., 1999; Tucker et al., 2005), especially in attacking-related indicators. In the CSL, home field advantage is also reported (Liu et al., 2019a; Zhou et al., 2019), although its long-term impact fluctuates (Zhou et al. 2020). However, the quality of the opposition is considered a stronger determinant than match location (Liu et al., 2016). Linked to this team’s ability, team budget is an indicator used to measure the strength of a team. Team budget has been proven to affect teams’ performance (Lago-Peñas and Gomez-Lopez, 2014; Lago-Peñas and Sampaio, 2015; Liu et al., 2019b) as richer teams cannot only afford the best coaches and players but also provide the best training and playing conditions and then adapt differently to new season performance (Lago-Peñas and Gomez-Lopez, 2014). Hence, the higher the budget the teams scoring first have, the greater their winning probabilities (Gómez-Ruano et al., 2013). Moreover, the influence of the first scoring time or match period on match performance (García-Rubio et al., 2015; Lago-Peñas et al., 2016; Ibáñez et al., 2018; Martinez and Gonzalez-Garcia, 2019) was also studied, but these results are still contradictory and need further investigation.

In modern sports, statistical and machine learning models are increasingly incorporated in the analysis of football games, which helps both researchers and practitioners better and more deeply understand game performance and its important influencing events (e.g., goals, scoring, fouls, etc.) and, hence, change the path to success. In particular, regression models (García-Rubio et al., 2015), classification trees (Lago-Peñas et al., 2016) and Cox models (Nevo and Ritov, 2013) were used to evaluate the effect of scoring first on match outcomes. Compared with early studies focusing on frequency, descriptive analysis, and comparisons (Yiannakos and Armatas, 2006; Armatas et al., 2009a; Leite, 2015), machine learning models can handle larger datasets and nonlinear relationships and then offer more knowledge to researchers (Hucaljuk and Rakipović, 2011; Liu, 2014; Liu et al., 2018).

Studies on match analysis in the Chinese Football Super League (CSL) have received considerable attention starting a few years ago because of its fast progress. These research studies focus primarily on key performance indicators (Mao et al., 2016; Yang et al., 2018; Gai et al., 2019), the impact of situational variables (Liu et al., 2019b), the evaluation of performance (Zhou et al., 2018, 2020), and the application of new technologies (Han et al., 2020) and complex networks (Yu et al., 2020; Zhao and Zhang, 2020). However, very few researches on the influence of key match events on CSL matches currently exist. Therefore, the present study investigates the impact of the first goal on the match outcome (win, draw, and lose) and the goal difference (GD) in the Chinese Super League. Thus, three hypotheses are proposed: (a) scoring first increases a team’s winning probability, (b) the first goal occurring later in a match increases a team’s winning probability, and (c) the influence of the minute of the first goal and goal difference on the match outcome is also influenced by situational factors and the team’s budget.

## Materials and Methods

### Data Sources and Reliability

Technical performance data were collected from the website of Champion,^1^ a leading Chinese sport data company. The validity and reliability of the Champdas Master Match Analysis System was verified by Gong et al. (2019), who demonstrated that Aiken’s *V* averaged 0.84 ± 0.03 and 0.85 ± 0.03 for the validation of indicators. Moreover, high kappa values (Operator 1: 0.92 and 0.90; Operator 2: 0.91 and 0.88), high intraclass correlation coefficients (varied from 0.93 to 1.00), and low standardized typical errors (varied from 0.01 to 0.34) indicated a high level of intraoperator reliability. The kappa values for the interoperator reliability were 0.97 and 0.89.

Preseason budget data (transfer value) were collected *via* a website,^2^ which was previously employed by Liu et al. (2019b). A positive correlation has been shown between the so-called “market values” determined by players’ salaries and the teams’ ranking (Bryson et al., 2013).

### Samples and Variables

From the 1,200 matches played in the 2014 to 2018 seasons in the CSL, a final sample of 1,116 matches in which at least one goal was scored were observed. The CSL is the highest-level national football league in China, and it includes 16 teams. These teams played in a balanced home and away schedule (15 home and 15 away matches for each team and season).

The dependent variables (Table 1) that were used were match outcome (MO) and the absolute goal difference (AGD). The independent variables were as follows: time (T), match location (ML), team budget (TB), and opponent budget (OB). In order to avoid estimation bias caused by endogenous variables, TB and OB were measured according to the rank of the preseason budget, considering that the preseason budget has a great association with the end-of-season ranking in football (Lago-Peñas and Sampaio, 2015; Liu et al., 2019b). All the observed criteria were applied to the team that scored first.

**Table 1:** Description and categories of the variables.

Variables	Description
Match outcome (MO)	Outcome at the end of the match. It can end in a draw or in a victory for the local or visiting team
Absolute goal difference (AGD)	Goal difference at the end of the match
Time (T)	Time (in minutes) where the first goal is scored
Match location (ML)	Team playing like local or visiting
Team budget (TB)	Budget of the team scoring first (budget high or low)
Opponent budget (OB)	Budget of the opponent to the team scoring first (budget high or low)

### Procedure and Statistical Analysis

First, after data were collected, they were ordered in specific spreadsheets, and information about match location, minutes when the goal was scored, and team budget was added. The teams were clustered into two groups in terms of the team’s budget (high or low budget) using a k-means (Schwartz’s Bayesian) technique. The higher group had a mean budget of 50.77 million dollars, while the lower group had a mean of 13.77 million dollars.

Second, the descriptive statistics (means and standard deviations) were calculated for the match outcome (MO) vs. match location (ML) and team budget (TB).

Third, in order to estimate the effects of the minute of the first goal on the match outcome, a multinomial logit regression model was adopted. The model can be specified as follows:

(1) $$\begin{array}{l} MO_{ij\left(win,draw,lose\right)}=\alpha_{0j}+\alpha_{1j}Time_{ij}+\alpha_{2j}ML_{ij}+\\ \;\;\;\;\;\;\;\;\;\;\;\;\;\;\;\;\;\;\;\;\;\;\;\;\;\;\;\;\;\;\;\;\;\;\;\;\;\;\;\;\;\;\;\;\;\;\;\;\;\;\;\alpha_{3j}TB_{ij}+\alpha_{4j}OB_{ij}+\varepsilon_{ij} \end{array}$$

where *Y_ij_* is a random variable indicating the result of the ith match (j ∈ (1,2,3), where 1 = *win*, 2 = *lose*, and 3 = *draw*), *α*_0*j*_ is the constant terms, and *ε_ij_* is the disturbance terms that are independent and identically distributed following a Gumbel (type 1 extreme value) distribution. The equation shows the influences of the independent variables related to scoring first on match outcome.

Additionally, a Poisson regression model was applied to further investigate the effects of scoring first on the absolute goal difference:

(2) $$\begin{array}{l} AGD_{i}=\beta_{0}+\beta_{11}Time_{i}*D_{1}+\beta_{12}Time_{i}*D_{2}+\beta_{21}ML_{i}*\\ \;\;\;\;\;\;\;\;\;\;\;\;\;\;\;\;\;\;\;\;\;D_{1}+\beta_{22}ML_{i}*D_{2}+\beta_{31}TB_{i}*D_{1}+\beta_{32}TB_{i}*D_{2}+\\ \;\;\;\;\;\;\;\;\;\;\;\;\;\;\;\;\;\;\;\;\;\beta_{41}OB_{i}*D_{1}+\beta_{42}OB_{i}*D_{2}+\mu_{i} \end{array}$$

where *β*_0_ is the constant term, and *μ_i_* is the error term for this regression model. *D*_1_ and *D*_2_ are dummy variables indicating whether a first scoring team eventually won the match. *D*_1_ = 1 if the first team that scored won the match, and *D*_2_ = 1 if the first team that scored did not win the match. Thus, the coefficients of the interaction terms in model (2) show the impacts of the variables related to scoring first on the absolute value of the goal difference, conditional on the match outcome for the first scoring team (i.e., either win or not win).

The abovementioned models were estimated using STATA for Windows (version 15.0, Stata Corp., Texas, United States), and the level of significance was set at *p* < 0.05.

## Results

As Table 2 shows, the majority of teams that scored first won the games (66.31%), followed by achieving a draw (20.70%) and losing (12.99%). Regarding the match location, the teams who scored first and played at home mostly won the matches (71.94%), followed by achieving draws (19.97%) and losing (8.99%). On the other hand, the teams who scored first and played away won the match in 58.60% of the cases, achieved a draw in 22.93% of the cases, and lost in 18.47% of the cases. Additionally, in terms of the team budget, when high-budget teams scored first, they won a higher percentage of their matches (76.10%) than low-budget teams (63.47%), whereas low-budget teams drew more matches (21.85%) than high-budget teams (16.75%).

**Table 2:** Description of the game results of the scoring first teams.

	Win	Draw	Lose	Total
**Match location**				
Home	464 (71.94%)	123 (19.97%)	58 (8.99%)	645
Away	276 (58.60%)	108 (22.93%)	87 (18.47%)	471
**Team Budget**				
Budget high	191 (76.10%)	42 (16.73%)	18 (7.17%)	251
Budget low	549 (63.47%)	189 (21.85%)	127 (14.68%)	865
Total	740 (66.31%)	231 (20.70%)	145 (12.99%)	1,116

The estimation results of the multinomial logit model are presented in Table 3. “Lose” was the omitted category (the basic outcome) with which the estimated coefficients are compared. The relative risk ratios are also included in this table to make the interpretation easier. All estimated parameters were statistically significant, indicating that the match location and team budget were the main factors.

**Table 3:** Estimation results of the multinomial logit model.

Variables	***β*** Coefficient	Std. Err.	Relative-risk ratios	Marginal effects
***Lose***				Pr = 0.1299
Time of the first scoring	(Base outcome)			−0.0022^***^
Match location				−0.0952^***^
Team budget				−0.0975^***^
Opponent budget				0.0647^***^
Constant				
***Win***				Pr = 0.6631
Time of the First Scoring	0.0232^***^	0.0049	1.0234	0.0028^***^
Match location	1.0083^***^	0.1910	2.7409	0.1342^***^
Team budget	1.0534^***^	0.2721	2.8674	0.1490^***^
Opponent budget	−0.6568^***^	0.2240	0.5185	−0.0752^**^
Constant	0.4254	0.1892	1.5303	
***Draw***				Pr = 0.2070
Time of the first scoring	0.0155^***^	0.0055	1.0156	−0.0006
Match location	0.5892^***^	0.2170	1.8026	−0.0390
Team budget	0.5501^*^	0.3072	1.7334	−0.0514^*^
Opponent budget	−0.4748^*^	0.2588	0.6220	0.0106
Constant	−0.1840	0.2154	0.8319	
N. of Obs.		1,116		
Log likelihood		−923.2150^***^		
LR chi2		81.15^***^		

The base outcome in this multinomial logit model is “Lose.” “Pr” is the predicted probability calculated at sample mean value.

* Significance levels are indicated as *p* < 0.1.

** Significance levels are indicated as *p* < 0.05.

*** Significance levels are indicated as *p* < 0.01.

In order to better understand the effects of the selected variables, the marginal effects of changing their values on the probability of observing the three match outcomes were, respectively, calculated. The values of the predicted probabilities indicated that the first scoring teams in the sample had the highest probability of winning (66.31%) and the lowest probability (12.99%) of losing matches. The probability of a draw was 20.70%. A statistically significant impact of the time of the first goal in the match-on-match outcome was found. The positive signs of its coefficients indicated that the later the goal scored, the higher the probabilities of winning or drawing. Moreover, the calculated average marginal effect demonstrated that postponing the first goal for 1 min increased the average probability of winning by 0.0028 while decreasing the probability of losing by 0.0022. Match location and the team budget of the first team that scored had a significant influence on game results. If the high budget team scores first playing at home, there were greater probabilities of winning or drawing than losing. Concretely, compared with away teams, home teams were approximately 13.42% more likely to win and approximately 9.52 and 3.90% less likely to lose and draw, respectively. High-budget teams were approximately 14.90% more likely to win and approximately 9.75 and 5.14% less likely to lose and draw, respectively, than low-budget teams.

The estimation results of the Poisson regression model are reported in Table 4. Most estimated coefficients in this model were statistically significant. To help interpret the parameter estimates of the Poisson regression, the marginal effects of all dependent variables for the probabilities of having a goal difference in observed matches were also listed.^3^ The minute of the first goal had negative effects on the absolute value of the goal difference. For any team scoring first, the earlier the first goal was scored, the larger the goal difference. Moreover, match location positively affected the goal difference. The opposite signs of the two interaction terms of match location and the dummies (*D*_1_ and *D*_2_) indicated that for the teams scoring first who won the match in the end, home teams had a higher likelihood of obtaining a final score with a larger goal difference than visiting teams. Furthermore, for those who did not win the match, home teams have a higher likelihood of obtaining a final score with a smaller goal difference than visiting teams. Besides, among the first scoring teams that won the match, teams with a high budget were more likely to obtain a final larger goal difference than those with a low budget.

**Table 4:** Maximum likelihood estimation (MLE) results of the Poisson regression model.

Variables	Coef.	Std. Err.	Marginal effects for Pr (AGD >= 1)
Time for the first scoring * D1	−0.0044^*^	0.0012	−0.0012^*^
Time for the first scoring * D2	−0.0289^*^	0.0034	−0.0084^*^
Match location * D1	0.2287^*^	0.0558	0.0666^*^
Match location * D2	−0.7314^*^	0.1292	−0.2129^*^
Team budget * D1	0.2704^*^	0.0581	0.0787^*^
Team budget * D2	−0.5874	0.2045	−0.1710
Opponent budget * D1	−0.0352	0.0744	−0.0102
Opponent budget * D2	0.2382	0.1397	0.0693
Constant	0.5149^*^	0.0567	
N. of Obs.	1,116		
Log pseudolikelihood	−1,463.3526^*^		
Wald chi2	56.21^***^		

* Significance levels are indicated as *p* < 0.01.

**Figure 1:**
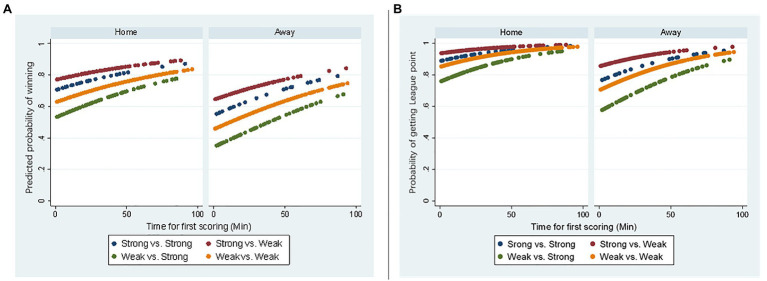
Predicted probability of winning a match **(A)** or getting league points **(B)**.

The predicted probabilities of winning a match and obtaining league points for the first scoring teams were also graphed. At a given minute for the first goal, home teams had a higher probability of winning than away teams, while high-budget teams had a higher probability of winning than low-budget teams (Figure 1A). Furthermore, for any team, as the minute of the first goal moves forward to the end of the match, the predicted probability of winning increased accordingly. Furthermore, the team scoring first showed a high probability (>50%) of obtaining league points (Figure 1B).

## Discussion

The aim of this study was to explore the effect of the minute of the first goal on the match outcome. The results found that (a) scoring first has a positive impact on the match outcome in the CSL. The teams scoring first have an 87.01% probability of being unbeaten and a probability of winning five times than of losing in the league. (b) The time of the first score in a match has a statistically significant impact on match outcomes. Delaying the first minute score for 1 min increases the average probability of winning for the first scoring team by 0.0028. (c) The impact of scoring first is also affected by the match location (ML) and team budget (TB). Thus, the higher the budget of the team that plays at home, the greater the probability of winning the match and achieving a higher goal difference if its first goal is scored at the beginning of the match. Therefore, the impact of scoring first is affected by contextual factors, such as the match location and team budget. The three hypotheses proposed are accepted.

The current research states that the influence of scoring the first goal in CSL was strong. Of all matches with scoring, 66.31% of the teams that scored first won their matches. Additionally, in all 1,200 matches, 80.92% of the teams that scored first won or drew at the end of the match. When playing at home, first scoring teams have an approximately 80% likelihood of obtaining league points and an over 50% win probability. When playing on the road, these results are over 50% and approximately 40%, respectively. This trend of the advantage of scoring first was consistent with previous results on both international and national events. In international tournaments (including FIFA World Cup), scoring first helped teams win over 70% of their matches (Leite, 2015; Vergonis et al., 2019). In domestic leagues, scoring first also increased the probability of winning (71.43–86.15%; Armatas, et al., 2009a,b; Molinuevo and Bermejo, 2012; Lago-Peñas et al., 2016). In the CSL, the 66.31% probability of winning for teams scoring first is less than that of listed tournaments, which is probably because at the event level, high-level tournaments such as the World Cup and UEFA Champions League have stronger teams. Scoring even one goal in a match between strong teams might be decisive, which possibly results in a high advantage of scoring first.

This advantage of scoring first was traditionally associated with tactical, technical, physical, and psychological factors. In football matches, those teams scoring first tend to change their playing style and strategy after scoring a goal (Bloomfield et al., 2005; Taylor et al., 2008). In order to prevent their opponent from scoring, they usually adopt a more conservative strategy (more defensive; Lago-Peñas and Gomez-Lopez, 2014). Previous research also found that some of the performance indicators of teams scoring first are similar to successful teams (Szwarc, 2004). The reason could be that after the first goal is scored, players’ psychological status changes. Compared with their opponents’ pressure score after conceding a goal, a goal makes them feel relatively at ease in the following periods of the match and increases their confidence (Theis, 1992). Especially when playing at home, home teams enjoy home crowd support (Pratas et al., 2018), which negatively affects away teams (Wolfson et al., 2005). While scoring first improves a team’s confidence, it also negatively impacts its opponent’s psychology, consequently reducing its opponent’s performance in competition (Leite and Barreira, 2014).

The regression model also reveals the following: (1) match outcome (win, draw, and loss) is influenced by the time of the first score (time), match location (ML), team budget (TB), and opponent budget (OB); and (2) the absolute goal difference (AGD) is influenced by the time of the first score (time), match location (ML), and team budget (TB). The later the first goal is made, the clearer the match outcome is determined. As stated in the *Introduction* section, in a low-scoring sport, the first goal is influential in determining the match result (Bloomfield et al., 2005; Lago-Peñas et al., 2016). In addition, a case study of Real Betis (a La Liga club) conceding goals reports that the last 15 min is decisive in the balance of goals conceded, and this influence increases if the team is losing (Méndez et al., 2020). Nevo and Ritov (2013, p.175) suggested that “if a team is leading or behind by a goal, their next goal is equally likely to be scored. While time passes with no goal being scored, the probability of a goal in the next move is [*sic*] increases.” The current study found that it is partially true that the earlier the first goal of the team is, the larger the absolute goal difference (AGD). However, it depends on team strength and where the match is held (match location). If a team that has a high budget and plays at home scores first, it is very likely to continue scoring and win the match.

However, if a team that has a low budget and is playing in the road scores first at a very early time, this team may concede more goals through the match. These findings are consistent with Pratas et al. (2018) who found that in the Portuguese Premier League, a first scoring home team would easily score a second goal according to the first scoring time. For an away team, having an advantage (specifically only one goal) will not naturally lead to a victory because the home team will not give up easily, especially when the home team is down by only one goal at halftime (Heuer and Rubner, 2012). Nevertheless, home field advantage is not the same for all teams (Clarke and Norman, 1995). In the Chinese Super League, strong and weak teams have different home field advantages. A long-term study (Zhou et al., 2020) found that strong opponents become continuously difficult to beat, which is probably due to their increasing budgets and the investment of rich CSL clubs. Thus, the home field advantage appears in matches with a balanced quality between teams, whereas unbalanced matches are favorable to the strong team regardless of the match location (Liu et al., 2019a). In the Spanish women’s football professional league, the best teams are also highly effective regardless of the match location (Ibáñez et al., 2018). As a result, home field advantage is more likely to play a role in “plus value.”

Applied in previous studies (Lago-Peñas and Gomez-Lopez, 2014; Lago-Peñas and Sampaio, 2015; Liu et al., 2019b), team budget was proven to have a major influence on teams’ performance. Team budget indicates that rich clubs more easily sign star players and consequently organize a strong team. Good players are good in their physical (e.g., more high-intensity running, etc.; Mohr et al., 2003), tactical, and technical skills and their psychological ability against pressure (Lago-Peñas and Sampaio, 2015). These playing abilities help them adapt to the match situation and perform well and steadily on the pitch, even when facing a match status of losing. Moreover, players in successful teams perform more effectively and have higher quality of the tactical and technical skills rather than the number and quantity (Lago-Peñas et al., 2010; Yue et al., 2014; Liu et al., 2015). This could be the reason that strong teams can usually pull back when losing in an early stage in the match, and even they are able to get a big win in the end. However, weak teams need to change their strategies because they are not “strong” enough to keep their playing style under different situations. Furthermore, Mohr et al. (2003) reported that a reduced amount of high-intensity work toward the end of the match was linked to the match outcome. Strong teams have better physical conditioning, ensuring that they maintain high-quality technical and tactical actions (e.g., passing accuracy) and make fewer errors toward the end of match regardless of whether they are winning or losing. However, when weak teams are losing, their need for scoring strengthens their players’ anxiety, resulting in increased offensive action but more errors (García-Rubio et al., 2015). This could be seized by strong teams, leading to conceding goal(s).

Despite these findings, the main limitation of this study is related to the small number of variables examined, the lack of information about technical-tactical performance indicators leading to goals, and substitutions or players’ exclusion during games. Moreover, CSL clubs have increased their budgets and investment in recruiting talented players over these years, but this had not occurred for every club; and the long-term influence needs to be noticed. Last, team budget and match location might have interacted to influence match results, which needs to be further considered. Future studies are suggested in order to merge all the contextual variables (especially the quality of the opposition) with the team budget and the main players signed by teams at the beginning of the season.

## Conclusions and Practical Applications

This study demonstrates that there is a strong advantage when a team scores first in the CSL. Even low-budget teams maintain a high probability of winning (63.47%). For home teams and high-budget teams, they have a very high probability of winning a game (home team 71.94% and high-budget team 76.10%) when they score first. The time of scoring the first goal has an impact on the match outcome, and scoring first at a later stage increases the win probability. The impact of scoring first is also influenced by contextual variables. When low-budget teams or visiting teams score first, they must focus more on their defense, or they may be more likely to lose. When high-budget teams or home teams score first, they tend to continue to score more. When high-budget teams or home teams concede a goal first, especially in the early phase of a match, they still have chance to equalize the score and even score many more goals to win. These results may help coaches develop proper strategies when facing different match statuses and different qualities of opposition in terms of team budget according to the match location.

## Data Availability Statement

Publicly available datasets were analyzed in this study. This data can be found at: http://data.champdas.com.

## Author Contributions

TL and QC conceptualized the study. TL, AG-d-A, and QC contributed to the methodology and reviewed and edited the manuscript. QC contributed to the software, data collection, and visualization. TL wrote and prepared the original draft. HW and PH helped in improving this work. All authors contributed to the article and approved the submitted version.

### Conflict of Interest

PH was employed by company Microsoft, Beijing.

The remaining authors declare that the research was conducted in the absence of any commercial or financial relationships that could be construed as a potential conflict of interest.

## Footnotes

^1^http://data.champdas.com

^2^http://transfermarkt.de

^3^The marginal effects of the explanatory variables for the probabilities of zero goal difference (in other words, the probabilities for the first scoring teams drawing) have the same magnitudes but opposite signs. Considering the main purpose of this paper, the following discussion will focus primarily on the probabilities of having a goal difference (i.e., the cases when the first scoring teams won or lost the match).
